# Comparison of Characteristics and Inpatient Outcomes of Patients With Inflammatory Bowel Disease and Colon Cancer: A Propensity-Based Nationwide Inpatient Sample Study

**DOI:** 10.7759/cureus.20186

**Published:** 2021-12-05

**Authors:** Prashant Subedi, Mukesh S Paudel, Vijay Gayam, Amrendra Mandal

**Affiliations:** 1 Internal Medicine, Patan Academy of Health Sciences, Kathmandu, NPL; 2 Gastroenterology, National Academy of Medical Sciences, Kathmandu, NPL; 3 Gastroenterology and Hepatology, The Brooklyn Hospital Center, Brooklyn, USA; 4 Internal Medicine/Gastroenterology, State University of New York (SUNY) Upstate Medical University, Syracuse, USA

**Keywords:** national inpatient sample database, mortality, morbidity, colon cancer, inflammatory bowel disease

## Abstract

Introduction

Patients with inflammatory bowel diseases (IBDs) frequently develop colon cancer. Previous studies have identified the association between IBD and colon cancer. In this study, we explored the characteristics and outcomes of IBD patients with colon cancer admitted to the hospitals of the United States.

Methods

Patients who were hospitalized patients with diagnoses of IBD and colon cancer were compared with patients with IBD without colon cancer. The data were extracted from the Nationwide Inpatient Sample (NIS) from January 2016 to December 2017. Comparisons were made with regards to mortality, complications, in-hospital stay, and cost of treatment between the two groups.

Results

We identified 1,82,025 hospitalizations from January 2016 to December 2017 admitted with a diagnosis of IBD. Of these, 181,560 patients had IBD without colon cancer, and 465 patients had IBD with colon cancer. No statistically significant difference was observed with regards to the in-hospital mortality between the two groups. There were higher odds of acute kidney injury (AKI) (OR 1.54, 95% CI 6.6-9.8; p=0.00), colectomy (OR 1.2, 95% CI 1.3-2.5; p=0.0) and lower gastrointestinal bleeding (LGIB) (OR 1.6, 95% CI 1.8-3.7; p=0.04) in patients with IBD and colon cancer. A longer length of stay (7.1±6.9 vs.5.0±5.6, p=0.00) and higher mean total charge ($20,283 vs. $12,166, p=0.00) were observed in patients with IBD with colon cancer.

Conclusions

Patients with IBD-associated colon cancer appear to have higher complication rates, higher costs, and more extended hospital stays. Therefore, early identification and management of complications related to IBD among patients with colon cancer are particularly crucial to reduce morbidity as well as the cost of hospitalization and treatment.

## Introduction

Inflammatory bowel disease (IBD) is a chronic inflammatory condition of the bowel which is characterized by exacerbations and remissions and also a higher risk of colon cancer [1]. The incidence of IBD in the United States ranges between 2.2 and 19.2 cases per 100,000 person-years [2,3]. The most important and well-recognized risk factors identified for IBD-associated colorectal cancer are disease duration and extent. Chronic inflammation is believed to promote carcinogenesis [4]. Moreover, patients with IBD have a higher incidence of colon cancer and death as compared to the general population [4,5].

There have been remarkable developments in the treatment strategy of IBD in the last 20 years in the form of colonoscopy surveillance, and the use of biologics [6]. The North American consensus statement recommends regular monitoring of the large bowel with colonoscopy every one to two years in those with a disease duration of eight years [3]. Initiation of biological agents in these patients has altered the natural course of the IBD and has transformed the treatment perspective in the last two decades [5,7]. The genetic factors coupled with the chronic inflammatory activity in the colon of IBD patients are hypothesized to take part in a significant role in carcinogenesis, and influencing the inflammatory process could lower this continuous process of inflammation to dysplasia and eventually the development of carcinoma [4,5].

In the present Nationwide Inpatient Sample (NIS) study, we aimed to assess the length of stay (LOS), hospital cost, morbidity, and mortality of patients with IBD and colon cancer in the United States. This article was previously presented as an e-poster at the 2020 American College of Gastroenterology Annual Scientific Meeting on October 23-28, 2020.

## Materials and methods

This was a retrospective study. The data were collected from January 2016 to December 2017 Nationwide Inpatient Sample. The database is contributed by the Healthcare Cost and Utilization Project (HCUP) which is a family of databases including the State Inpatient Databases (SID), the NIS, the Kids' Inpatient Database (KID), and the outpatient databases State Ambulatory Surgery Data (SASD) and State Emergency Department Data (SEDD). After 2015, the NIS began to utilize the International Classification of Diseases, Tenth Edition, Clinical Modification/Procedure Coding System (ICD-10-CM/PCS) [8].

This study was exempted from the Institutional review board considering the unrecognizable coded data. We analyzed the data of patients older than 18 years of age using ICD-10 codes. The data were classified into two groups: patients with IBD and colon cancer and patients with IBD without colon cancer. The patient demographics such as age, race, and sex; the Charlson Comorbidity Index, insurance status, hospital characteristics, and relevant comorbidities were tabulated.

The primary outcome of the study was all-cause in-hospital mortality. Secondary outcomes comprised of the incidence of sepsis, acute kidney injury (AKI), colectomy, peritonitis, intestinal perforation, fistula, intestinal obstruction, and lower gastrointestinal bleeding (LGIB). Complications were recognized using their particular ICD-10-CM/PCS. We also studied the LOS, factors affecting LOS, and average hospital costs.

We conducted all statistical analyses as per the recommended methods accounting for the intricate survey design of the NIS database [9]. Categorical data were reported as frequency and percentage, and continuous data as mean with standard deviation and standard error. Categorical variables were analyzed using Pearson's chi-square test, and continuous variables were analyzed using the Student's t-test. Unadjusted odds ratios for the primary and secondary outcomes were calculated using univariate logistic regression. Multivariable logistic regression was used to adjust for potential confounders in the final model. Statistical significance was set at a two-sided p-value of <0.05. STATA/MP 15.10 (StataCorp LLC, College Station, TX, USA) was used for statistical analysis. All analyses in our study were weighted using provided discharge weights to produce national estimates. Hospital costs were inflation-adjusted for 2018 using the Consumer Price Index (provided by the US Department of Labor).

The propensity score matching was applied to analyze the difference in the baseline as well as outcomes [10,11]. We added covariates in the model and also incorporated covariates in the final model if the coefficient changed by more than 20%.

## Results

We identified 182,025 hospitalizations from 2016 to 2017 admitted with the diagnosis of IBD. Of these, 181,560 had IBD without colon cancer, and 465 had IBD with colon cancer. The mean age of patients in the IBD with colon cancer group was 42±18.9 years and in IBD without colon cancer group was 54±14.9 years. Before propensity matching, there was a statistically significant difference observed with regards to age, race, discharge disposition, hospital region, teaching hospital status, and Charlson co-morbidity index between the two groups. There was also a significant difference with regards to co-morbidities, which included anemia and chronic kidney disease (CKD) as shown in Table 1.

**Table 1:** Comparison of demographic parameters between patients of IBD with colon cancer and IBD alone *Denotes parameters with a statistically significant difference; SD: standard deviation; DM: diabetes mellitus; CHF: congestive heart failure; HTN: hypertension; CAD: coronary artery disease

Variable	IBD without colon cancer	IBD with colon cancer	P-value
Total	181,560	465	
Age in years±SD	42±18.9	54±14.9	0.00*
Female (%)	52.8	46.2	0.20
Race			0.00
Caucasian (%)	72.1	76.9	
African American (%)	14.6	10.9	
Hispanic (%)	8.5	2.2	
Asian (%)	1.4	4.4	
Native American (%)	0.4	3.3	
Others (%)	2.8	2.2	
Hospital Bed Size			0.4
Small	18.3	23.6	
Medium	27.8	26.8	
Large	53.8	49.4	
Hospital Region (%)			0.03*
Northeast	21.3	20.4	
Midwest	23.9	32.2	
South	38.4	25.81	
West	16.2	21.5	
Type of Discharge			0.04*
Routine	83.4	64.5	
Skill Nursing Facility	1.4	2.1	
Charlson Co-morbidity Index (%)			0.00*
0 or 1	87.8	20.0	
2	6.3	34.0	
3	5.8	45.9	
Insurance Type (%)			0.18
Medicare	23.3	26.8	
Medicaid	21.4	15.0	
Private	49.6	55.9	
Uninsured	5.5	2.1	
Teaching Hospital	72.8	82.8	0.03*
Chronic Co-morbidity			
DM (%)	8.6	12.9	0.14
CHF (%)	2.2	3.2	0.51
Anemia (%)	10.7	17.2	0.04*
HTN (%)	21.7	27.9	0.14
Obesity (%)	8.3	8.6	0.91
Dyslipidemia (%)	11.3	13.9	0.41
CAD (%)	4.9	7.5	0.25
Chronic Kidney Disease (%)	3.6	10.7	0.003*

All covariates from Table 1 were used to construct a propensity score. A propensity score matching model was developed to derive two matched groups for comparative outcome analysis from accounting for potential confounding factors and reducing the effect of selection bias. We analyzed the matched cohorts for covariate balance. As shown in Table 2, matching removed almost all significant differences.

**Table 2:** Balance of covariates after propensity score matching *Denotes statistically significantly different results; DM: diabetes mellitus; CHF: congestive heart failure; HTN: hypertension; CAD: coronary artery disease

Covariates	IBD without colon cancer	IBD with colon cancer	P-value
Mean age (years)	57.5	55.6	0.2
Female (%)	47.2	46.1	0.8
Race			0.11
Caucasian (%)	77.8	77.4	
African American (%)	12..3	10.9	
Hispanic (%)	1.0	2.1	
Asian (%)	3.9	4.3	
Native American (%)	2.9	3.2	
Others (%)	2.1	2.1	
Hospital Bed Size (%)			0.38
Small	20.3	22.6	
Medium	27.8	26.8	
Large	51.8	50.4	
Hospital Region (%)	18.3	23.6	0.26
Northeast	21.4	21.1	
Midwest	32.9	31.8	
South	24.8	25.2	
West	20.9	21.9	
Discharge			0.80
Routine	80.3	79.4	
Skill Nursing Facility	2.1	2	
Charlson Co-morbidity Index (%)			0.65
0 or 1	34.3	31.2	
2	25.6	26.9	
3	40.1	41.9	
Insurance Type (%)			0.31
Medicare (%)	21.1	22.8	
Medicaid (%)	22.3	21.9	
Private (%)	50.2	51.1	
Uninsured (%)	6.4	4.2	
Teaching Hospital (%)	79.1	82.4	0.57
Chronic Co-morbidity			
DM (%)	12.1	12	0.90
CHF (%)	3.1	3.0	0.91
Anemia %)	19.7	17.0	0.5
HTN (%)	28.5	28.5	1.00
Obesity (%)	7.0	8.2	0.5
Dyslipidemia (%)	14.2	15.5	0.5
CAD (%)	7.6	8.2	0.6
CKD (%)	11.0	12.0	0.81

We compared the outcomes of IBD with colon cancer vs. IBD without colon cancer. There was no statistically significant difference observed with regards to in-hospital mortality either before propensity matching score (OR 2.0, 95% CI 0.36-1.2; p=0.4) or after propensity matching score (OR 1.3, 95% CI 0.89-1.6; p=0.32) as shown in Table 3.

**Table 3:** Comparison of primary and secondary outcomes: IBD with colon cancer versus IBD alone *Denotes results with a statistically significant difference; IBD: inflammatory bowel diseases; AKI: acute kidney injury; LGIB: lower gastrointestinal bleeding

	Before propensity score matching			After propensity score matching		
Variable	Odds ratio	P-value	95% CI	Odds ratio	P-value	95% CI
In-hospital Mortality	2.0	0.4	0.36-1.2	1.3	0.32	0.89-1.6
Sepsis	1.1	0.66	0.28-3.4	1.3	0.56	0.38-2.5
Peritonitis	1.07	0.9	0.22-5.0	1.12	0.8	0.78-2.2
AKI	1.01	0.4	0.38-1.5	1.54	0.00*	6.6-9.8
Colectomy	4.6	0.00*	2.6-8.1	1.2	0.00*	1.3-2.5
Blood Requirement	1.05	0.9	0.46-2.3	1.01	0.56	0.46-3.7
Intestinal Perforation	1.22	0.7	0.29-5.0	1.14	0.34	0.75-.25
Intestinal Obstruction	1.22	0.6	0.47-3.1	1.9	0.42	0.33-1.2
Intestinal Fistula	1.9	0.11	0.84-4.5	1.3	0.22	0.87-7.8
LGIB	1.01	0.04*	1.3-2.5	1.6	0.04*	1.8-3.7

In the unmatched cohort, there were higher odds of colectomy (OR 4.6, 95% CI 2.6-8.1; p=0.00) and LGIB (OR 1.01, 95% CI 1.3-2.5; p=0.04) in the IBD with colon cancer vs. IBD without colon cancer. After matching the cohorts, there was higher odds of AKI (OR 1.54, 95% CI 6.6-9.8; p=0.00), colectomy (OR 1.2, 95% CI 1.3-2.5; p=0.0) and LGIB (OR 1.6, 95% CI 1.8-3.7; p=0.04).

There was a significant statistical difference observed in the mean LOS and the mean total charge between the two groups. Longer LOS (7.1±6.9 vs.5.0±5.6, p=0.00) and higher mean total charge ($20,283 vs. $12,166, p=0.00) were observed in the IBD with colon cancer when compared to the IBD without colon cancer (Table 4).

**Table 4:** Analysis for the length of stay and cost of care *Denotes result with a statistically significant difference; IBD: inflammatory bowel diseases; LOS: length of stay

	IBD without colon cancer (%)	IBD with colon cancer (%)	P-value
Mean LOS (Days)	5.0±5.6	7.1±6.9	0.00*
Mean Total Charge ($)	12,166	20,283	0.00*

The factors affecting LOS in IBD with colon cancer were intestinal perforation (95% CI, 1.6-3.6; p=0.008), peritonitis (95% CI, 2.6-4.7; p=0.00), sepsis (95% CI 1.5-2.1; p=0.00), diabetes mellitus (95% CI1.5-4.5; p=0.04), and dyslipidemias (95% CI 2.5-6.6; p=0.007) (Table 5).

**Table 5:** Factors affecting the length of hospital stay CI: confidence interval; DM: diabetes mellitus

Variables	P-value	CI
Intestinal perforation	0.008	1.6-3.6
Peritonitis	0.00	2.6-4.7
Sepsis	0.00	1.5-2.1
DM	0.04	1.5-4.5
Dyslipidemia	0.007	2.5-6.6

## Discussion

In this large retrospective study using the NIS size representing 182,025 admissions, we analyzed the outcomes of patients of IBD with colon cancer and compared them with those of patients of IBD without colon cancer. There was no statistically significant difference observed with regards to the in-hospital mortality rate between the two groups. However, the study showed worse outcomes, such as AKI, colectomy, and LGIB, in IBD with colon cancer patients. There was also higher resource utilization, including, LOS, and total hospitalization costs in patients with IBD and colon cancer. The factors affecting the LOS in IBD with colon cancer were intestinal perforation, peritonitis, sepsis, diabetes mellitus, and dyslipidemias.

Previous studies have demonstrated the rising incidence of IBD over the past several years in the general population worldwide. Still, to our knowledge, there are no previous studies related to the NIS database regarding the outcome of IBD in a patient with colon cancer [3,12-15]. Some studies also showed as many as 18% of people with IBD might develop colorectal cancer after the development of IBD [16]. The increased risk of colon cancer has been associated with the duration of IBD, the segment of the colon involved as well as the severity of the IBD. Due to this risk associated with IBD, patients are recommended to undergo more frequent colonoscopies than the average risk population [17]. This recommendation has resulted in decreasing the incidence of colon cancer in IBD patients, which was observed in a previous large study [13].

The rate of colectomy in our study is supported by the observational study by Navaneethan et al. study who also demonstrated that colectomy rates among IBD patients with colon cancer are significantly higher. Additionally, they also showed a decrease in the trend of colectomy rate annually declining from 1995 to 2012 in the USA [18].

Our study did not show a significant difference in in-patient mortality between the two groups. However, a population-based cohort study with five years follow-ups has shown a higher mortality rate-ratio in patients with IBD and colon cancer as compared to those with IBD alone [19]. This difference can be explained by the lack of follow-up data in our study. The risk of colorectal cancer increases with younger age of diagnosis, the extent of involvement of colon with IBD, and longer duration of symptoms. It has been shown that 5%-10 % of IBD patients develop colon cancer after 20 years of diagnosis [20]. We did not have data regarding the duration of symptoms and extent of IBD and hence this comparison could not be done.

The primary limitation of our study is the cross-sectional design and identified data is limited to in-patient stays only, and thus they are also subject to misclassification. The NIS considers each hospitalization as a separate entry, so it is not possible to separate index cases from readmission, resulting in an overestimation of the number of admissions. Also, the potential of the inaccuracy of ICD-10-CM diagnostic coding in the database and that only in-patient discharge data representative of hospitals participating in HCUP. Information about medications is not included in the NIS.

## Conclusions

The present study demonstrated an increased rate of complication and considerably higher healthcare costs, and more extended hospital stay among patients with concomitant IBD and colon cancer. Therefore, early identification and management of complications related to IBD among patients with colon cancer are particularly crucial to reduce hospital costs and morbidity.

Future prospective studies using long-term follow-ups will be necessary to interpret the influence of the evidence-based practice and surveillance plan of actions on general outcome and survival of patients with IBD and colon cancer.
